# Supplementary material for: Circulating IL-6, IL-10, and TNF-alpha and IL-10/IL-6 and IL-10/TNF-alpha ratio profiles of polyparasitized individuals in rural and urban areas of gabon
Source: PLoS Negl Trop Dis. 2022 Apr 14;16(4):e0010308. doi: 10.1371/journal.pntd.0010308 (PMC9041759; doi:10.1371/journal.pntd.0010308)
Supplement: S1 Table — Differences between median percentages of parasitaemia were determined by the Bonferroni test. a, b and c: Comparisons of median parasitaemia between patients with malaria only versus malaria/filariasis co-infection (p = 0.039), malaria/STH co-infection (p = 0.0047), and malaria/intestinal protozoa co-infection (p = 0.016). d and e: Comparisons of median parasitaemia between patients with malaria/filariasis co-infection versus malaria/STH co-infection (p = 0.4) and malaria/intestinal protozoa co-infection (p = 0.6). f: Comparison of median parasitaemia between patients with malaria/STH co-infection versus malaria/intestinal protozoan co-infection (p = 0.3). g and h: Comparison of median microfilaremia between patients with filariasis versus malaria/filariasis co-infection: p = 0.7 for L. loa and p = 0.3 for M. perstans. i E. h/d: E. histolytica/dispar. (DOCX) [file pntd.0010308.s001.docx]

**S1 Table:** Parasitological characteristics of the study groups. Differences between median percentages of the parasitaemia were determined by the Bonferroni test.

|  | No parasites | *P. falciparum* only | Filariasis | STH | Intestinal protozoa only | *Plasmodium*/filariae coinfection | *Plasmodium*/STH coinfection | *Plasmodium*/intestinal protozoan coinfection |
| --- | --- | --- | --- | --- | --- | --- | --- | --- |
| Malaria *falciparum,* T/µL median [IQR] | - | 9450 [1296 - 36400] | - | - | - | 350 [16 - 1400]^a^ | 42 [12.3 – 681.5]^b,d^ | 749 [35 - 10150]^c,e,f^ |
| *L. loa,* µf/mL median [IQR] | - | - | 1100 [175 - 5625] |  | - | 700 [250 - 3500]^g^ | - | **-** |
| *M. perstans,* µf/mL median [IQR] | - | - | 200 [100 - 450] | - | - | 650 [300 - 1000]^h^ | - | **-** |
|  | **N** | **N** | **N** | **N** | **N** | **N** | **N** | **N** |
| Blood filariasis (n=55) | - | - | 48 | - | - | 7 | - | - |
| *Loa loa* (n=51) | - | - | 46 | - | - | 5 | - | - |
| *Mansonella perstans* (n=10) | - | - | 8 | - | - | 2 | - | - |
| Intestinal helminths (n=56) | - | - | 5 | 43 | - | 1 | 7 | - |
| *A. lumbricoides* (n=35) | - | - | 2 | 29 | - | - | 4 | - |
| *T. trichiura* (n=33) | - | - | 3 | 26 | - | 4 | - | - |
| *S. stercoralis* (n=5) | - | - | - | 3 | - | 2 | - | - |
| *N. americanus* (n=1) | - | - | - | 1 | - | - | - | - |
| Intestinal protozoan (n=108) | - | - | 11 | 32 | 39 | 2 | 7 | 17 |
| *B. hominis* (n=87) | - | - | 7 | 28 | 32 | 1 | 5 | 14 |
| *E. coli* (n=41) | - | - | 4 | 17 | 11 | 1 | 6 | 2 |
| *E. h/d^i^* (n=17) | - | - | 3 | 6 | 4 | - | 3 | 1 |
| *G. intestinalis* (n=5) | - | - | - | 1 | 3 | - | 1 | - |

^a^,^b^ and ^c^ : Respectively comparisons of median parasitaemia between patients with malaria only *versus* malaria/filariasis coinfection (*p*=0.039), malaria/STH coinfection (*p*=0.0047), and malaria/intestinal protozoa coinfection (*p*=0.016). ^d^ and ^e^: Respectively comparisons of median parasitaemia between patients with patients with malaria/filariasis coinfection *versus* malaria/STH coinfection (*p*=0.4), malaria/intestinal protozoa coinfection (*p*=0.6). ^f^: comparison of median parasitaemia between patients with malaria/STH coinfection *versus* malaria/intestinal protozoan coinfection (*p*=0.3). ^g^ and ^h^: comparison of median microfilaremia between patients with filariasis *versus* malaria/filariasis coinfection: *p*=0.7 for *L. loa* and *p*=0.3 for *M. perstans*. ^i^ *E. h/d*: *E. histolytica/dispar.*
