# Supplementary material for: Circulating IL-6, IL-10, and TNF-alpha and IL-10/IL-6 and IL-10/TNF-alpha ratio profiles of polyparasitized individuals in rural and urban areas of gabon
Source: PLoS Negl Trop Dis. 2022 Apr 14;16(4):e0010308. doi: 10.1371/journal.pntd.0010308 (PMC9041759; doi:10.1371/journal.pntd.0010308)
Supplement: Attachment — Submitted filename: renamed_0081d.docx [file pntd.0010308.s002.docx]

**Responses to reviewers**

**Methods**
Reviewer #1:

-Are the objectives of the study clearly articulated with a clear testable hypothesis stated? YES
-Is the study design appropriate to address the stated objectives? YES
-Is the population clearly described and appropriate for the hypothesis being tested? No, it needs further clarification
-Is the sample size sufficient to ensure adequate power to address the hypothesis being tested? No, but given the characteristics of the work it is enough.
-Were correct statistical analysis used to support conclusions? Yes, although in some occasion it needs further explanation.
-Are there concerns about ethical or regulatory requirements being met? Yes, important official references are missing.

**Me: Reference and additional information were added in the “Ethical consideration” section line 315 “under the reference PROT No 003/2016/SG/CNE”. Also at lines 323-326 “The study was explained to the population. In the informed consent form, it is indicated that the patient agrees that these samples be used for other research purposes. All samples were analyzed with the approbation of participants during the main study where samples were collected”**

Further comments follow:
- Figure 1: This figure needs some remodeling and clarification from authors. The box with the final number of patients selected for the study (n=240) is currently named “patients selected randomly”. This is not true, as the samples were not selected randomly but after exclusion criteria showed right above it. The “molecular diagnosis of malaria” box in the middle should be renamed, as all samples are not diagnosed using molecular techniques. I suggest authors change it to just “diagnosis of malaria”. More importantly, numbers do not match. In theory there are 72+5=77 plasmodium positive samples (as per the diagnosis of malaria box); however, in the last row there are 50+7+7+17=81 samples that have plasmodium (alone or in combination). This mismatch applies to the whole manuscript and table 1 as well.

**Me: all these corrections were taken into account in the figures below at the line 155. Four patients were tested positive for the malaria with rapid diagnosis test. There were not considered in the previous version. But with these we can have the number of 81.**

- L295-297: This information either needs to be referenced to the appropriate section where they are explained, or directly moved to those sections.

**Me: The four first line of the “DNA extraction and submicroscopic plasmodial infection” section were insert from the L295-297 of the old version of the manuscript. “After the microscopic diagnosis of malaria, blood samples of those with negative blood smears were screened for submicroscopic plasmodial infection by nested polymerase chain reaction after DNA extraction.**

**Precisely,”.**

Reviewer #2: The main concern about the study is the lack of a clear hypothesis. This lack is highlighted by the confused conclusion presented by the authors. In addition, the results presented in the manuscript did not show any significant correlation worthy of note.

**Me: The main hypothesis of the article is that coendemic parasites can play a role in the malaria susceptibility of Gabonese populations. This is why in the results we present comparisons of the cytokines level and cytokines ratio between patients with mono and coinfection. Then to highlight the hypothesis that older children and adults are more infected by other parasites than young children and that this can be a reason of the shift of the at risk population and the increase of the number of cases in these age groups, in the results section, we show cytokine production according to age group and to parasitic profile.**

**Not significant correlations were observed in this paper, but it was important to publish these data to try to have other topics of research on coinfection and to show that intestinal protozoa are important in our context when we use cytokines ratio as indicator of malaria susceptibility.**

Line 45, not even all negative blood smears means absence of plasmodial infection that could be evidenced by microscopic evaluation of smears. I really didn't understand what the authors were supposed to mean by “submicroscopic plasmodial infection”. The authors should revise the use of “submicroscopic” terminology trough manuscript (line 76).

**Me: We define susbmicroscopic infection in a new section “definition” at lines 410-412. We supposed that if two readers examined a slide, it is enough to conclude to a negative result. So if DNA of Plasmodium sp was detected after nested PCR, we supposed that this patient had a infection not detectable at the microscopy. This is why the term submicroscopy plasmodial infection was used through the manuscript.**

Line 51, p=0.09 is correct?

**Me: Yes, it is**.

In abstract section, it was not clear what was the main objective of the study. There is not a clear conclusion as well.
**Me: “The aim of the study is to compare the cytokines level and cytokines ratio according to parasitic profiles of the population to determine the potential role of the co-endemic parasites in the malaria susceptibility of populations” was added to the abstract section at lines 42-44.**
--------------------

**Results**
Reviewer #1: -Does the analysis presented match the analysis plan? YES
-Are the results clearly and completely presented? NO (see below)
-Are the figures (Tables, Images) of sufficient quality for clarity? No, they need improvement, further details and clarification

Further comments:
- Table 2 needs further explanation in the legend and in the text. What are the P values in the last column referred to? In other words, what comparisons were made to obtain the P values shown (all groups together, infected vs uninfected, control vs a particular group…)?

**Me: these remarks were taken into account in the legend of the table 2 at lines 197-200: “The Kruskal-Wallis H-test allowed comparing all groups together. P-values of this comparison were presented in the last column of the table. The second column untitled “No parasites” indicates the group control that allows observing an increasing/decreasing of the cytokines level compared to other groups. Values obtained by the Kruskal-Wallis H-test were presented opposite:”.**

- Figure 2: In order to make the figure more attractive visually and easier for the reader to understand, I suggest authors only show statistical differences between groups. Currently it is packed with information almost overlapping, making it hard to read and interpret.

**Me: No significant correlations were observed in this manuscript. So this is why all p-values were conserved in the figure 2. Further explanations were given in the legend of the figure 2 at lines 224-225: “The p-value considered significant for analysis presented in this graphic is 0.000357. All the associations showed none significant difference.”**

- L167-169: Please rephrase, it is hard to understand what authors try to convey in this sentence.

**Me: lines 178-181: “The proportions of individuals with P. falciparum/STH co-infections and P. falciparum/intestinal protozoan co-infections were higher in younger children aged of less than 5 years old (50.0%) and those aged between 5 and 15 years old (52.9%) respectively”**

- L213-215: These sentences are confusing and are sending contradictory messages.
**Me: lines 229-231: “After Bonferonni correction in the figure 4a and considering a p-value <0.000357, no significant relationship was found. So, in the paragraph below, will be presented only comparisons with p-values nearest of the significance”**

Reviewer #2: The results presented in the manuscript did not show any significant correlation worthy of note.
**Me: as say previously, a not significant result can be important for the following research of our Department of Parasitology. We have put in evidence the supposed role of intestinal protozoa in the malaria susceptibility in the same way as helminths. Due to the small sample size, we cannot say it but, this is a new topic to explore not well known in parasitology. More is known on the role of the intestinal protozoa in the modification of the intestinal microbiota but this implies also a modification of the host immune system.**
--------------------

**Conclusions**
-Are the conclusions supported by the data presented?
-Are the limitations of analysis clearly described?
-Do the authors discuss how these data can be helpful to advance our understanding of the topic under study?
-Is public health relevance addressed?

Reviewer #1: -Are the conclusions supported by the data presented? YES
-Are the limitations of analysis clearly described? YES
-Do the authors discuss how these data can be helpful to advance our understanding of the topic under study? YES
-Is public health relevance addressed? YES
No further comments for this section.

Reviewer #2: In conclusion section, the authors stated that STH would be protective against malaria while intestinal protozoa may have a detrimental effect, based only in cytokines levels ratio, however, this is not enough to affirm that. The study not has sufficient data to achieve this conclusion.

**Me: We think that this is important to inform the scientific community that intestinal protozoa can modulate the host immune response. But it is true that we need a larger sample size as added in lines 550-551: “but there is a need to investigate this hypothesis with a larger sample size”. Or another option is to carry out a case/control study where PBMC will be collected in carrier of intestinal protozoa or not, and stimulated cells with plasmodial antigens.**

Still in conclusion, the authors stated that children with more than 5 years old and adults seem to be more at risk of infection when co-infected with filariasis associated with a higher IL-10/TNF-α ratio, and when co-infected with intestinal parasites associated with a higher IL-10/IL-6 ratio. It is not clear what the authors wanted sated here, but this sentence not make it any sense at all. Maybe the authors would mean that co-infected people should be more susceptible to present clinical symptomatology or bad outcome in case of infection with P. falciparum.
**Me: yes, we can say that. This is why in lines 554-555 we added “This can suppose that co-endemic parasites can also explain the observed epidemiological transition of malaria with shift of at risk population”.**
--------------------

**Editorial and Data Presentation Modifications?**
Reviewer #1: - Author’s summary section needs significant remodeling and English editing. Currently it is hard to understand on account of incomplete sentences, wrong choice of words or the lack of a clear link between ideas in different sentences, among others. For instance, in L80-81 it is not clear what authors try to convey here: helminths are not protozoan parasites but they seem to be considered like that.

Me: at different levels of this section, we improved the editing.

**Lines 70-79: The current epidemiological transition of malaria observed in Gabon, had for consequence the shift of the at risk population of the children aged of less than 5 years old to older children aged of 5-15 years. Another consequence was the number of the increasing number of cases of infection in adults among adults. It is important to explain this malaria rebound. Intestinal parasites and blood filariasis are endemic in Gabon. These parasites are described to alter the malaria immune response and can be implicated in the susceptibility of individuals to malaria. In Gabon, malaria presents a heterogeneous repartition. Thus, in the present study, we investigate the role of co-endemic parasitosis in the alteration”**

**Lines 81-90: “ of malaria. Microscopic analyses were performed for the parasitological diagnosis. The nested-PCR technique was used to put in evidence the submicroscopic parasitaemia of *Plasmodium* sp. Then, once groups with different parasitological profiles were constituted, IL-6, IL-10 and TNF-α were measured in the plasma of individuals. Th2/Th1 ratios, which can indicate the level of susceptibility of individuals to malaria, were calculated. We observed that there is no interaction between *Plasmodium* sp and co-endemic parasites in the present study. However, the high level of Th2/Th1 cytokines ratio among patients with intestinal protozoa seem suggest that these intestinal parasites could also play a role in the susceptibility to malaria as helminths”**

- In general, the manuscript needs a thorough and deep English editing, as currently it is hard to follow on many parts due to wrong choice of words, missing or wrong prepositions and incomplete sentences, among others.

**Me: We have contacted a professional in the translating and submitted the manuscript for the improvement of the English editing. Unfortunately, we have not yet received the revised version. This is the reason I submitted at the last minutes the revised version. This is also why in the e-mail of the 08/10/2021, I asked 7 additional days to submit a revised version of good quality in the English editing. The English editing was corrected by Springer Nature service. The English editing was done after all reviewers corrections. This version was uploaded. So you will find 2 manuscripts and 2 manuscripts with changes highlighted.**

- Figures and tables need some further clarification and other minor modifications (see above)
**Me: please see responses above.**

Reviewer #2: (No Response)

--------------------

**Summary and General Comments**
Reviewer #1: In this work authors explore the influence of diverse co-infection with asymptomatic patients from Gabon by analyzing cytokine profiles and comparing the different groups composing their samples. Despite being relatively simple, the work is well carried out and conclusions are supported by the results, which can further advance in the knowledge of the devastating disease caused by Plasmodium. In general authors did a good job in all sections although there are many parts that need some editing, further clarification or more details. Importantly, English quality is deficient and a thorough and significant editing is needed before the manuscript can be acceptable for publication, as currently many parts are hard to follow or catch the message authors try to convey. Nevertheless, I believe this work has the potential to be published in PNTD should authors address the issues raised in this revision.

Reviewer #2: In this manuscript “Circulating IL-6, IL-10, TNF-alpha and IL-10/IL-6, IL-10/TNF-alpha Ratios Profiles of Polyparasitized Individuals in Rural and Urban Areas of Gabon”, the authors performed the quantification of cytokine levels of plasma of individuals from rural and urban area from seven sites of Gabon with different co-parasitism. The main concern about the study is the lack of a clear hypothesis. This lack is highlighted by the confused conclusion presented by the authors. In addition, the results presented in the manuscript did not show any significant correlation worthy of note. I explain my concerns in more detail below.
Major
In conclusion section, the authors stated that STH would be protective against malaria while intestinal protozoa may have a detrimental effect, based only in cytokines levels ratio, however, this is not enough to affirm that. The study not has sufficient data to achieve this conclusion.
Still in conclusion, the authors stated that children with more than 5 years old and adults seem to be more at risk of infection when co-infected with filariasis associated with a higher IL-10/TNF-α ratio, and when co-infected with intestinal parasites associated with a higher IL-10/IL-6 ratio. It is not clear what the authors wanted sated here, but this sentence not make it any sense at all. Maybe the authors would mean that co-infected people should be more susceptible to present clinical symptomatology or bad outcome in case of infection with P. falciparum.
**Me: Please see responses to these comments above.**

Minor
Line 45, not even all negative blood smears means absence of plasmodial infection that could be evidenced by microscopic evaluation of smears. I really didn't understand what the authors were supposed to mean by “submicroscopic plasmodial infection”. The authors should revise the use of “submicroscopic” terminology trough manuscript (line 76).

**Me: Please see the response in previous paragraphs**

Line 51, p=0.09 is correct?

**Me: Yes, it is**

In abstract section, it was not clear what was the main objective of the study. There is not a clear conclusion as well.

**Me: the main objective was added in lines 42-44 : “The aim of the study is to compare the cytokines level and cytokines ratio according to parasitic profiles of the population to determine the potential role of the co-endemic parasites in the malaria susceptibility of populations”**

Sentence in line 66-68 is confused, please revise it.
**Me: please see the corrections above.**


Figure Files:
While revising your submission, please upload your figure files to the Preflight Analysis and Conversion Engine (PACE) digital diagnostic tool, https://pacev2.apexcovantage.com. PACE helps ensure that figures meet PLOS requirements. To use PACE, you must first register as a user. Then, login and navigate to the UPLOAD tab, where you will find detailed instructions on how to use the tool. If you encounter any issues or have any questions when using PACE, please email us at [figures@plos.org](mailto:figures@plos.org).

**Me: I have used PACE. From Powerpoint I create images then I uploaded them in PACE. After downloaded them, I copied them in the main manuscript but they appeared in green. So the old versions of the images were conserved in the submitted manuscript.**
